# Minimization of the Settling Time of Variable Area Flowmeters

**DOI:** 10.3390/s19030530

**Published:** 2019-01-27

**Authors:** Mateusz Turkowski, Artur Szczecki, Maciej Szudarek

**Affiliations:** Warsaw University of Technology, Faculty of Mechatronics, Institute of Metrology and Biomedical Engineering, ul. Sw. Andrzeja Boboli 8, 02-525 Warszawa, Poland; m.turkowski@mchtr.pw.edu.pl (M.T.); szczecki@mchtr.pw.edu.pl (A.S.)

**Keywords:** variable area flowmeters, rotameters, optimization, flow sensors, industry 4.0, modelling

## Abstract

In the article a differential equation describing transient behavior of variable area (VA) meters has been developed and validated experimentally for air as a measured fluid and for two float shapes—plumb bob and sphere. A modified version of simplex algorithm adapted for nonlinear constraint optimization problems was applied to minimize the settling time of VA meters in two cases. In the first case both the float and tube geometry were altered. In the second case only the float geometry was modified. The second case has been validated experimentally. The theory and experiment is in reasonable agreement (under 5% of full scale), which is satisfactory for the purposes of optimization of VA flowmeters dynamic performance. Analytical model of VA flowmeter has been proven to be a proper tool for optimization. Settling times obtained during the optimization process were several times shorter than these of commercially manufactured instruments. Overshoot has not exceeded the assumed value of 3%.

## 1. Introduction

Over the recent decades various versions of variable area (VA) flowmeters have grown to maturity. E.g., classic “see through” rotameters, orifice VA meters or VA flow transducers. Both the calibration procedures [1,2] and corrections for density and viscosity [3,4] have been established for a long time. Many float types have been thoroughly investigated [5].

The simplest indicating device is a transparent tube. More sophisticated flow transducers use a magnetically inert metal tube. In this case the float is provided with a permanent magnet and float position is sensed by an external magnet. Float displacement can then be converted to any standardized signal. Simple solution consists of a linear variable differential transformer [6]. Another possibility is the application of Hall sensors [7,8], magnetoresistive sensors matrix parallel to the tube [9] or capacitance transmitters [10]. In this case transducer does not exert forces on the float and therefore does not influence the VA meter dynamics. This simple and economical flowmeter continues to be one of the most widely used instruments for low and medium flow rates in numerous industry branches [11,12].

Industry 4.0, which is the current trend of automation and data exchange in manufacturing technologies including cyber-physical systems and Internet of Things, favors measurement sensors which have good dynamic performance [13], e.g., Coriolis flowmeters. However, price of Coriolis flowmeters is one or two orders of magnitude higher than price of simple VA flow transmitters. Study on dynamic performance of VA meters would allow optimization and development of simple and rugged flow transmitters that can meet contemporary needs of process automation.

Up till now research on dynamics of variable area flowmeters has concerned their behavior in presence of pulsations [14,15,16]. This aspect is important in case of such pulsation sources as reciprocating pumps or other sources classified in Ref. [17]. However, for control purposes such parameters as settling time, nature of step response (periodic or aperiodic) and overshoot are vital. These subjects have not yet been studied in the context of VA meters. The paper deals with these aspects and presents optimization procedures. An analytical model describing VA meter dynamics is developed and validated. The methodology bases on registering float position by a camera. This is the only method that does not exert forces on the float. The proposed equation has been proven to work for air and for two float shapes—plumb bob and sphere. Further research is required to validate the developed equation for other fluids and other float shapes. Computational fluid dynamics may be a viable tool for this purpose. In that case simulation validation criteria described in Ref. [18] may be applied. After validation of the transient equation the article present results of optimization for two cases. In the first case both the tube and float geometry were altered, in the second case only the float geometry was modified. The second case was validated experimentally and the presented methodology has been proven to be of scientific and practical significance.

## 2. Materials and Methods

### 2.1. VA Flowmeter Characteristics in Steady State

**Figure 1:**
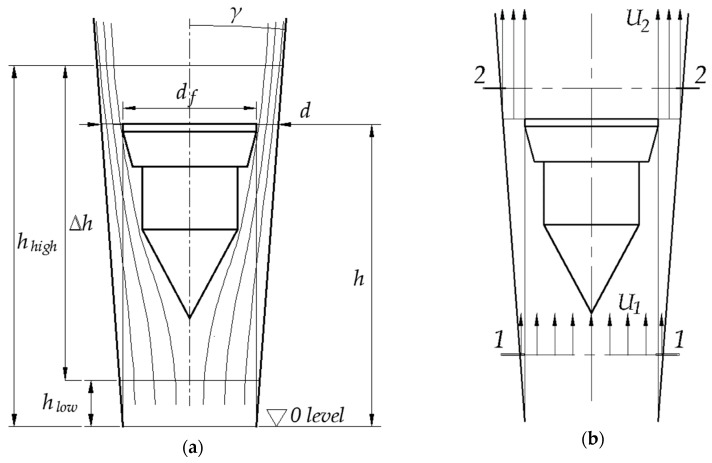
(**a**) Simplified geometry of a variable area flowmeter and the streamlines across it (not to scale); (**b**) Section 2.1. is where the float does not yet influence the flow. Section 2.2. is in the narrowest annular stream section, “vena contracta”. All of the marked heights are measured from the zero level. The zero level is the height at which diameter of the tube *d* is equal to the float diameter *d_f_.*

Variable area flowmeter consists of a tapered tube and a float. The float can take various forms. Examples are traditional plumb bob (as in Figure 1a), sphere or flat disc. Let us consider two perpendicular sections of the flowmeter marked in the Figure 1b. For stationary, incompressible flow one can use Bernoulli’s law to write:

(1) $$p_{1}-p_{2}=\frac{\rho }{2}\left(U_{2}^{2}-U_{1}^{2}\right)$$

where *ρ* is the fluid density and *p*_1_, *U*_1_, *p*_2_, *U*_2_ are respectively pressure and mean velocities in Section 2.1. and Section 2.2.

The area of the narrowest part of the stream *A*_2_ equals:

(2) $$A_{2}=C_{C}A$$

where *A* = π/4(*d*^2^ − *d_f_^2^)* and *Cc* is the contraction coefficient. As the velocity *U*_1_ is much smaller than *U*_2_, its square can be neglected. Therefore:

(3) $$U_{2}=\frac{Q}{C_{C}A}$$

(4) $$p_{1}-p_{2}=\frac{\rho }{2}{\left(\frac{Q}{C_{C}A}\right)}^{2}$$

this pressure difference multiplied by the area of the float *A_f_* = π*d_f_*^2^/4 results in active force directed upwards:

(5) $$F_{A}=A_{f}\left(p_{1}-p_{2}\right)=A_{f}\frac{\rho }{2}{\left(\frac{Q}{C_{C}A}\right)}^{2}$$

The restoring force *F_R_* is equal to the apparent weight of the float of volume *V_f_* and density *ρ_f_* immersed in the fluid of density *ρ*:

(6) $$F_{R}=gV_{f}\left(\rho_{f}-\rho \right)$$

where *g*—acceleration due to gravity. In the equilibrium state active force equals restoring force, therefore one can write:

(7) $$Q=C_{C}A\sqrt{\frac{2gV_{f}\left(\rho_{f}-\rho \right)}{\rho A_{f}}}$$

There are various methods to obtain *Cc* coefficient. Procedures of VA meter calculations based on *d*:*d_f_* diameter ratio and Ruppel number are shown in Ref. [2]. Another option is to determine *Cc* during calibration, as proposed by Ref. [1]. In this way a flow coefficient *C*(*Re*) is obtained, instead of the contraction coefficient *Cc*. Although *d*:*d_f_* is an important factor, it is taken here into account indirectly, through hydraulic diameter in *Re*. The flow coefficient *C*(*Re*) includes not only the effect of contraction, but also viscous friction, dynamic drag and other effects. In this case *Re* is defined as:

(8) $$Re=\frac{\left(d-d_{f}\right)U}{\nu }$$

where (*d* − *d_f_*) is hydraulic diameter, *U = Q*/*A*(*h*)—mean velocity in the annular slot between float and tube and *ν*—kinematic viscosity of the fluid.

### 2.2. VA Behavior During Transient

During transient the active force is proportional to the velocity relative to the float. Therefore, float velocity *dh*/*dt* must be subtracted from velocity *U*_2_ given by Equation (3). What is more, moving float acts as a piston pump. It results in an additional volumetric flow rate *Q*′ equal to:

(9) $$Q{}^{\prime }=A\frac{dh}{dt}$$

The resultant velocity equals:

(10) $$U{}^{\prime }=\frac{Q{}^{\prime }}{A_{2}}=\frac{A_{f}\frac{dh}{dt}}{C\left(Re\right)A}$$

taking the above into the account, mean velocity relative to the float in the Section 2.2. is:

(11) $$U_{2}(t)=\frac{Q}{C\left(Re\right)A}-\frac{dh}{dt}-A_{f}\frac{\frac{dh}{dt}}{C\left(Re\right)A}$$

the flow rate in the Section 2.2. equals:

(12) $$Q(t)=U_{2}(t)C\left(Re\right)A=Q-C\left(Re\right)A\frac{dh}{dt}-A_{f}\frac{dh}{dt}$$

and the active force during transient is then:

(13) $$F_{A}(t)=\rho A_{f}\frac{{\left(Q-C\left(Re\right)A\frac{dh}{dt}-A_{f}\frac{dh}{dt}\right)}^{2}}{2{\left(C\left(Re\right)A\right)}^{2}}$$

taking into account dynamics, the restoring force equals:

(14) $$F_{R}=gV_{f}\left(\rho_{f}-\rho \right)+m\frac{d^{2}h}{dt^{2}}$$

as *Q* can be in general a function of time *Q*(*t*), the equation of motion of the float will be therefore:

(15) $$m\frac{d^{2}h}{dt^{2}}=\rho A_{f}\frac{{\left[Q\left(t\right)-C\left(Re\right)A\frac{dh}{dt}-A_{f}\frac{dh}{dt}\right]}^{2}}{2{\left[C\left(Re\right)A\right]}^{2}}-gV_{f}\left(\rho_{f}-\rho \right)$$

It is easy to validate that in steady state (*h = const*.) this equation takes the form of static Equation (7). The relationship between the area *A* and the position of the float in the tube *h* (which is in fact the measure of the flow rate *Q*) is as follows:

(16) $$A(h)=\frac{\pi }{4}\left(d{\left(h\right)}^{2}-d_{f}^{2}\right)=\frac{\pi }{4}\left[{\left(d_{f}+2h\tan\gamma \right)}^{2}-d_{f}^{2}\right]=\pi \left(hd_{f}\tan\gamma +h^{2}\tan^{2}\gamma \right)$$

during transient Reynolds number equals:

(17) $$Re\left(t\right)=\frac{\left(d-d_{f}\right)U\left(t\right)}{\nu }$$

where:

(18) $$U\left(t\right)=\frac{Q-C\left(Re\right)A\left(h\right)\frac{dh}{dt}-A_{f}\frac{dh}{dt}}{C\left(Re\right)A(h)}$$

which allows to obtain the final equation:

(19) $$m\frac{d^{2}h}{dt^{2}}=\rho A_{f}\frac{{\left[Q-C\left(Re\right)A\left(h\right)\frac{dh}{dt}-A_{f}\frac{dh}{dt}\right]}^{2}}{2{\left[C\left(Re\right)A\left(h\right)\right]}^{2}}-gV_{f}\left(\rho_{f}-\rho \right)$$

The differential Equation (19) is nonlinear and it can be only solved numerically. Runge-Kutta algorithm has been used for this purpose. There was a question of approximating function *C*(*Re*), which has been obtained experimentally. After numerous attempts with polynomial, exponential and logarithmic curves the best fit was obtained using approximating function in form of:

(20) C(Re)=Dln[E⋅Re(t)+1]

where *D* and *E* coefficients were determined with the use of least squares method. As all the experiments were conducted with the use of atmospheric air as working medium, buoyancy forces could have been neglected. Therefore:

(21) $$m\frac{d^{2}h}{dt^{2}}=\rho A_{f}\frac{{\left[Q-C\left(Re\right)A\left(h\right)\frac{dh}{dt}-A_{f}\frac{dh}{dt}\right]}^{2}}{2{\left[C\left(Re\right)A\left(h\right)\right]}^{2}}-gm.$$

### 2.3. Validation of the Transient Equation

Seven VA flowmeters were investigated (Table 1). *d_low_* and *d_high_* are tube diameters at the heights corresponding to the lower and upper flow rate limits. Δ*h* is the distance between these extreme float positions (see Figure 1a). The tubes for VA flowmeter no. 6 and 7 are the same as for no. 2 and 4, only the float was replaced.

**Figure 2:**
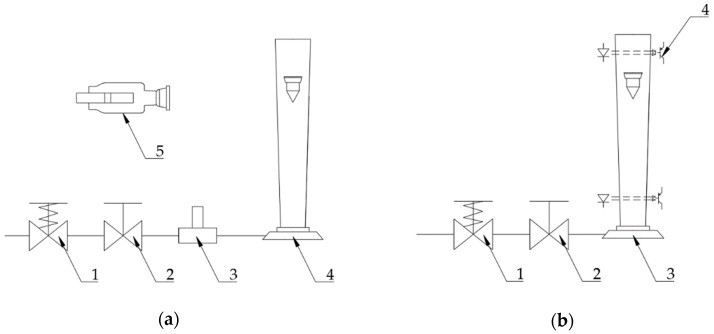
(**a**) Test stand for registration of the step response of VA flowmeter. 1—fast opening valve, 2—flow control valve, 3—constant temperature anemometer with data acquisition system, 4—VA flowmeter, 5—camera; (**b**) test stand for settling time measurement. 1—fast opening valve, 2—flow control valve, 3—VA flowmeter, 4—photoelectric sensors. Photoelectric sensors triggered a timer, enabling the measurement of the settling time.

The experiments were conducted with a methodology fully developed by the authors. The step response was filmed with an analog 8 mm camera with frame rate of 50 frames per second (Figure 2a). The position of the float was later measured on the screen of a film previewer. Step change in the flow rate was generated by a fast opening of a spring operated valve. 

**Figure 3:**
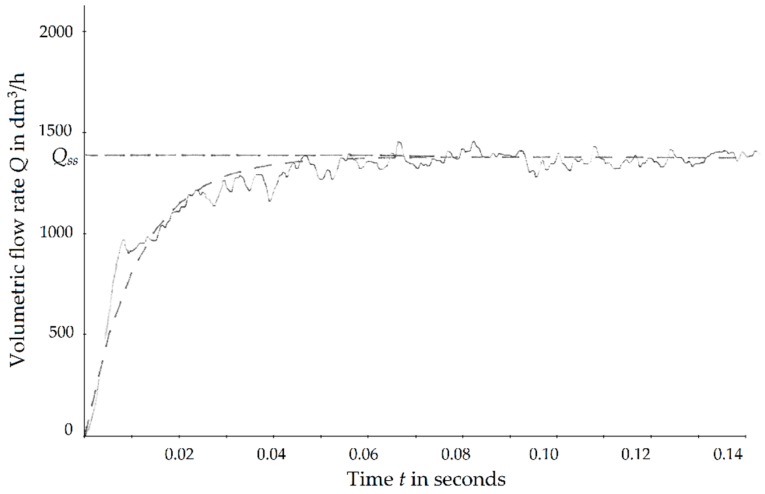
Step change registered by hot wire anemometer (solid line) and its approximation (dashed line).

The step was not ideal, therefore it has been registered by means of hot wire anemometer [19] and later approximated by a function in form of:

(22) $$Q\left(t\right)=Q_{SS}\left(1-e^{-90t}\right)$$

where *Q_SS_* is the flow rate in steady state. This form of excitation signal was used during the solution of the Equation (21). Exemplary signal registered by thermoanemometer and its approximation is shown in the Figure 3. Test stand for settling time measurement is presented in the Figure 2b. The movement of the float at the final phase was slow. For that reason the overshoot could have been easily assessed by visual observation.

### 2.4. Optimization

For a given flow rate range and measured fluid there are three parameters that define remaining parameters of a given VA flowmeter unambiguously. Examples are *d_low_*, *d_high_*, Δ*h* or *m_f_*, *d_low_*, Δ*h*. A set of parameters *d_f_*, *(d_low_* − *d_f_),* Δ*h* was chosen for the reason of simplicity of formulae to calculate the remaining parameters. A modified simplex algorithm described in the Appendix A was used for optimization. Considering the parameters of VA flowmeters manufactured commercially, constraints presented in the Table 2 were assumed.

The first three constraints are boundary constraints. As the density of float *ρ_f_* does not occur explicitly in the Equation (21), the 4^th^ constraint is referred to as inequality constraint. Volume of the float of a plumb bob type is expressed as *V_f_* = 0.679*d*^3^*_f_*.

**Figure 4:**
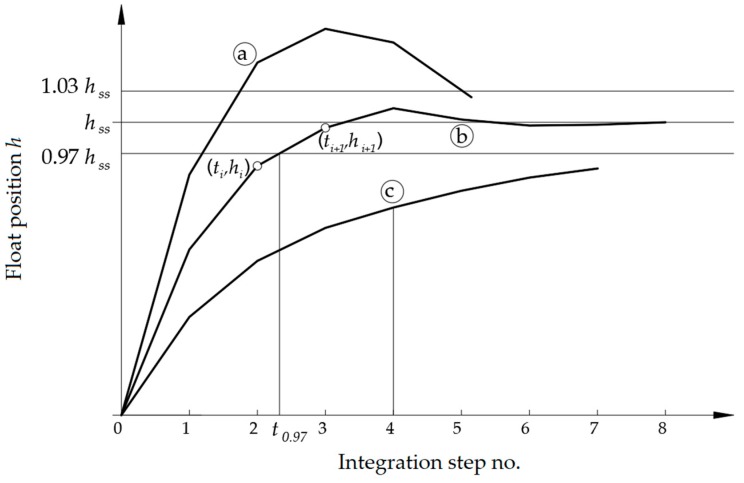
Definition of the objective function (graph not in a scale).

To determine values of coefficients *D* and *E* of the function approximating *C*(*Re*), all the experimental data concerning VA meters from Table 1 was collected. Function in a form of:

(23) C(Re)=0.1652ln[0.1537⋅Re(t)+1]

was determined with the use of least squares method and was used during optimization. From the point of view of automation systems, minimum settling time and an aperiodic response (possibly with one small overshoot) is advantageous. The objective function was therefore defined as in Figure 4. Optimized VA meters should have had a minimal settling time *t*_0.97_. The overshoot should have not exceeded 3% of the float position in a steady state *h_SS_*.

For the each set of chosen VA parameters generated randomly by optimization algorithm described in the appendix, the values of an objective function were obtained by integration of the Equation (21). After every step of integration (ca. 0.01 s) an analysis of the step response was performed. If the float height exceeded the value of 1.03*h_SS_* (point a) the integration was interrupted as not fulfilling the assumptions. The results were then rejected and a new integration process was started, based on a new set of parameters generated by the optimization algorithm. If the response did not exceed the 1.03*h_SS_* value and in subsequent integration step the calculated *h* value started to decrease (point b), it was no longer possible that the float went beyond the ±0.03*h_SS_* limits. The value *t*_0.97_ calculated as an abscissa of the intersection of the line connecting points (*t_i_*, *h_i_*) and (*t*_*i*+1_, *h*_*i*+1_) and the horizontal line *h* = 0.97*h_SS_* was stored for further processing by the algorithm. To save time, if after some arbitrarily chosen time the response did not reach 0.97*h_SS_*, the integration was interrupted (point c) and results were rejected. The optimization procedure stopped when five optimization results (objective function values) remained within a specified error band.

## 3. Results and Discussions

### 3.1. Validation of the Transient Equation

**Figure 5:**
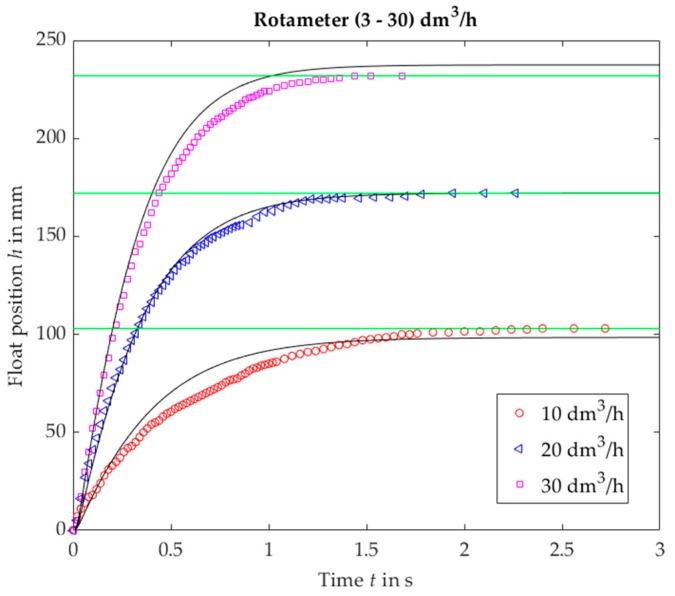
Rotameter no. 1, plumb bob. Float position *h* vs. time *t* for a step response, experimental (black solid lines) and calculated (markers).

**Figure 6:**
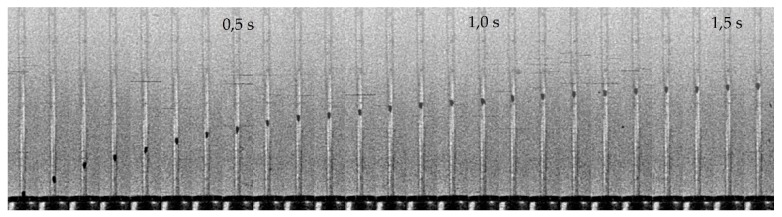
Rotameter no. 1, step 10 dm^3^/h. Selected images captured by a camera.

**Figure 7:**
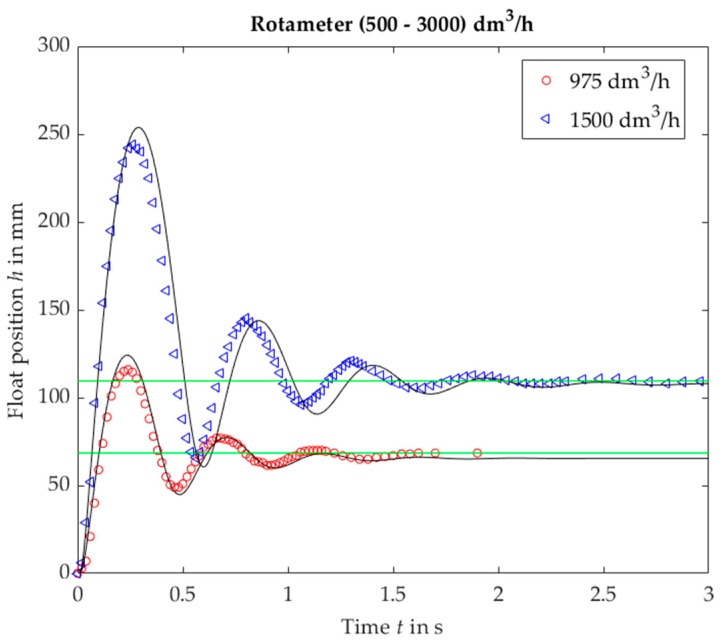
Rotameter no. 5, plumb bob. Float position *h* vs. time *t* for a step response, experimental (black solid lines) and calculated (markers).

**Figure 8:**
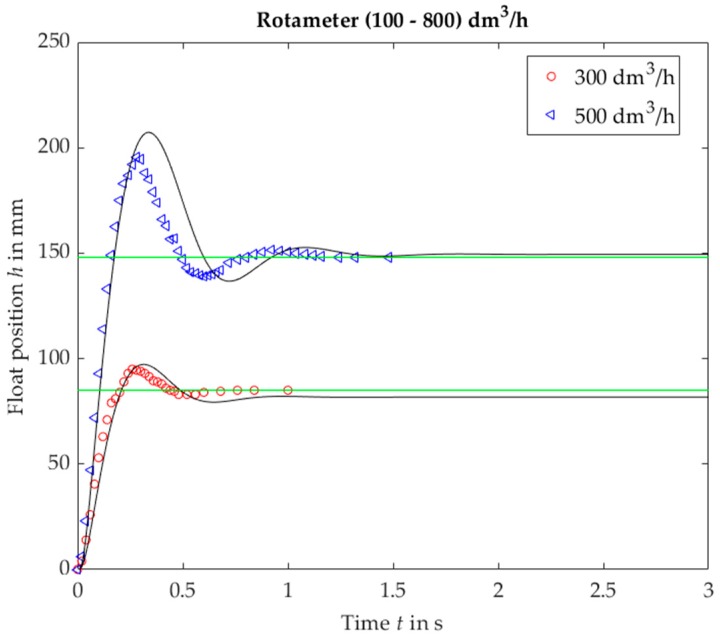
Rotameter no. 3, plumb bob. Float position *h* vs. time *t* for a step response, experimental (black solid lines) and calculated (markers).

**Figure 9:**
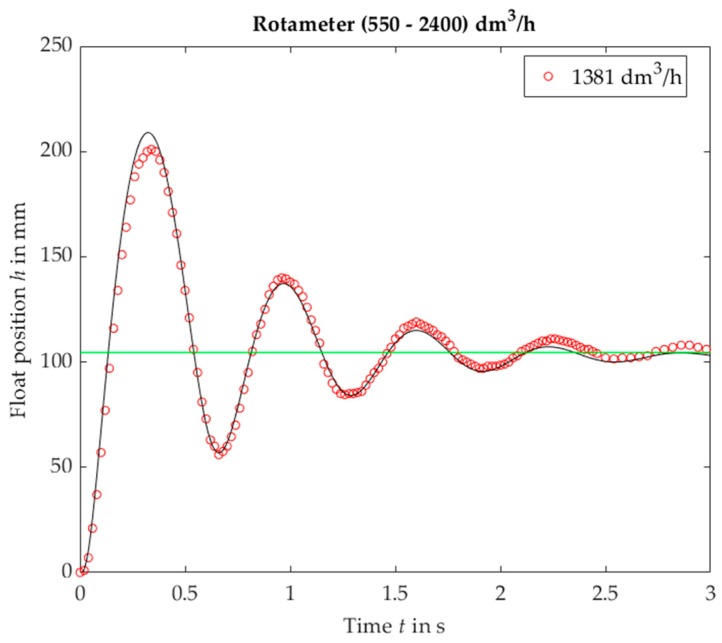
Rotameter no. 7, sphere shape. Float position *h* vs. time *t* for a step response, experimental (black solid lines) and calculated (markers).

**Figure 10:**
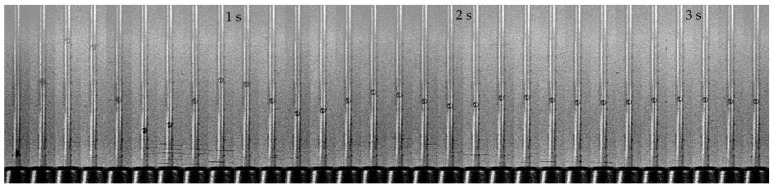
Rotameter no. 7. Selected images of a step response captured by a camera.

The comparison of experiments with the results obtained from the Equation (21) are presented in Figure 5, Figure 6, Figure 7, Figure 8, Figure 9 and Figure 10. One can distinguish between three typical step responses. For VA no. 1 an aperiodic response was obtained (Figure 5 and Figure 6). It resembles a step response of a first order linear system with damping coefficient above 1. The settling time rises with the decreasing value of step change, which is in contrast to a typical linear system.

For VA no. 5 results resemble a step response of a second order linear system with a small value of damping coefficient (Figure 7). As opposed to the linear system, the oscillation frequency is higher for a lower step value. Moreover, oscillation fragments below steady-state value are shorter than the upper parts. 

The response of VA no. 3 resembles a step response of a linear system with damping coefficient equal to ca. 0.5, practically with one overshoot (Figure 8). In contrast to the linear system, the overshoot decreases with a decreasing step value.

The response of VA no. 7 of a sphere shape (Figure 9 and Figure 10) is similar to the response of VA no. 5, but with a smaller value of damping coefficient.

Uncertainty analysis is shown in the Appendix B. The sum of the experimental and theoretical uncertainties is in the worst case equal to *U_95_*(*h*) = 18.2 mm whereas the differences between experiments and simulations were up to 11 mm. It was assumed that the mathematical model was in a satisfactory agreement with the experiments.

### 3.2. Optimization of VA Flowmeters

Three VA meters of a plumb bob type were optimized for nominal ranges listed in the Table 3. In studied cases the pipe length Δ*h* was equal to the lower bound *l_i_* = 150 mm and the overshoot was equal to 3% of *h_SS_* value. In comparison to the commercial VA meters, resultant float density was relatively low and the clearance between a float and a tube was relatively high. The settling time (0.32–0.35) s was shorter than for commercial VA meters (see Figure 5, Figure 6, Figure 7, Figure 8, Figure 9 and Figure 10 where it equals (0.9–1.5) s).

Unfortunately, unit production of glass tubes for experiments is costly. Therefore the optimization was repeated for the existing tubes specified in the Table 1, and was limited to modifying float parameters. In this case optimization algorithm generated the lower range limit *Q_min_* and the clearance (*d_low_* − *d_f_*). The remaining parameters such as upper range limit, float mass and density were then easily calculated. Additional restriction was added concerning the rangeability, namely *Q_max_*/*Q_min_* ≥ 8.

Optimization results are presented in the Table 4. Columns 4 and 6 contain values of the clearance (*d_low_* − *d_f_*) and *Q_min_* generated by the optimization algorithm. The calculated values of *ρ_f_* and *Q_max_* are presented in columns 5 and 7. Theoretical values of the settling time (column 8) are within the range of (0.341–0.372) s, which is also an improvement in comparison to the commercial meters. The experimental results are presented in columns 9–12. There is a good agreement between results of optimization and experiments. Measured settling times are equal or slightly shorter than theoretical values. The measured overshoot has never exceeded the assumed value of 3%.

## 4. Conclusions

Analytical model of VA flowmeters dynamics has been developed and validated experimentally. It has been proven to be a proper tool for optimization. This will make VA meters more appropriate for process automation, which is a prerequisite for the implementation of Industry 4.0 solutions. Settling times obtained during the optimization process were several times shorter than these of commercially manufactured instruments: (0.341–0.372) s instead of (0.9–1.5) s. This proves that the presented methodology is of scientific and practical significance. What is more, it has been proven that the developed equation describing VA meter dynamics is appropriate for at least two float shapes, i.e., plumb bob and sphere.

The sum of the experimental and theoretical uncertainties is in the worst case 18.2 mm whereas the differences between experiments and simulations were up to 11 mm. The discrepancies can also be attributed to the numerical modeling of the system (e.g., inaccurate physical models, discretization and round-off errors) and the fact that the uncertainty analysis has been developed for the steady state case, not for transient. What is more, the approximating function *C*(*Re*) that takes float shape into account has been obtained as the best fit of experimental data. Comparing the Figure 9 with Figure 5, Figure 7and Figure 8, it can be seen that the differences between modelling and experiments are smaller for a simple sphere float shape. However, the theory and experiment are in reasonable agreement (under 5% of full scale), satisfactory for the purposes of optimization of VA flowmeters dynamic performance. In generally, the discrepancies between the experiments and calculations are smaller for larger rotameters.

Further research should be conducted for various float shapes and various fluids as a measured medium. This includes both classical rotameters and variable orifice meters. The influence of forces from transmitter acting on a float should also be studied. It would also be beneficial to employ computational fluid dynamics for further optimization.

## Figures and Tables

**Table 1:** List of investigated VA flowmeters and their most important parameters.

VA no.	Nominal Range dm^3^/h	Float Shape	*d_f_* mm	*d_low_* mm	*d_high_* mm	Δ*h* mm	*m_f_* g	*D*	*E*
1	3–30	Plumb bob	3.990	4.080	4.310	236	0.053	0.2369	0.0815
2	20–220	Plumb bob	4.030	4.130	4.560	223	0.381	0.2485	0.0388
3	100–800	Plumb bob	8.910	9.307	10.360	216	0.595	0.2132	0.0502
4	200–2200	Plumb bob	9.020	9.237	10.354	215	4.055	0.1789	0.0929
5	500–3000	Plumb bob	11.030	11.22	12.485	236	7.298	0.1444	0.297
6	25–230	Sphere	3.972	4.130	4.560	223	2.54	0.2784	0.0447
7	550–2400	Sphere	8.731	9.237	10.354	215	2.79	0.1680	0.1654

**Table 2:** List of constraint functions for optimization problem.

No.	Lower Bound *l_i_*	Parameter	Upper Bound *u_i_*
1	4 mm	*d_f_*	25 mm
2	0.05 mm	*d_low_* − *d_f_*	1 mm
3	150 mm	Δ*h*	400 mm
4	0.2 g/cm^3^	*m*/(0.697*d*^3^*_f_*)	11 g/cm^3^

**Table 3:** Tube and float optimization results.

VA no.	Nominal Range in dm^3^/h	Float Shape	*d_f_* in mm	*d_low_* in mm	Tube Tapering	Δ*h* in mm	*ρ_f_* in g/cm^3^	*t*_0.97_ in s
1	10–100	Plumb bob	4.517	4.818	0.0031	150	0.437	0.349
2	50–500	Plumb bob	10.21	10.58	0.0066	150	0.258	0.336
3	200–2000	Plumb bob	20.53	20.88	0.0088	150	0.355	0.326

**Table 4:** Results of float optimization for tubes specified in the Table 1.

No.	Δ*h* in mm	*d_low_* in mm	*d_low_* − *d_f_* in mm	*ρ_f_* in g/cm^3^	Theoretical Values			Experimental Values			
					*Q_min_* in dm^3^/h	*Q_max_* in dm^3^/h	*t*_0.97_ in s	*Q_min_* in dm^3^/h	*Q_max_* in dm^3^/h	*t*_0.97_ in s	Overshoot in %*h_ss_*
1	2	3	4	5	6	7	8	9	10	11	12
1	236	4.080	0.091	4.098	7.5	73	0.372	8.2	62	0.35	2.0
2	223	4.130	0.106	1.235	2.6	68	0.341	3.2	64	0.31	1.9
3	216	9.307	0.339	0.299	37	361	0.341	46	384	0.32	2.1
4	215	9.237	0.381	0.252	38	352	0.345	37	361	0.33	2.3
5	236	11.22	0.402	0.252	60	554	0.356	72	566	0.36	1.1

**Figure A1:**
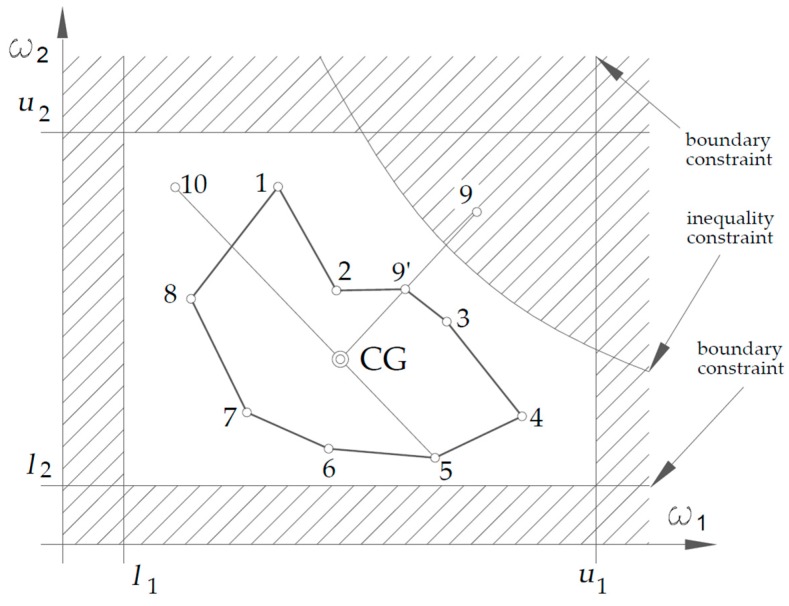
Scheme of the optimization procedure.

## Nomenclature

*A* = π/4(*d*^2^ − *d_f_*^2^) (m^2^)	Area of the annulus between tube and float
*A_2_* = *C_c_·A* (m^2^)	Area of the narrowest part of the stream
*A_f_* = π*d_f_*^2^/4 (m^2^)	Area of the float
*c_i_*	Sensitivity coefficient
*C* (-)	Flow coefficient
*C_C_* (-)	Contraction coefficient
*d* (m)	Tube diameter corresponding to the current float postition
*d_f_* (m)	Float diameter
*d_high_* (m)	Tube diameter at the height corresponding to the upper flow rate limit *Q_max_*
*d_low_* (m)	Tube diameter at the height corresponding to the lower flow rate limit *Q_min_*
*D*, *E* (-)	Coefficients of a curve approximating *C*(*Re*)
*F_A_* (N)	Active force
*F_R_* (N)	Restoring force
*g* (m/s^2^)	Acceleration due to gravity
*h* (m)	Position of the float related to the zero level
*h_ss_* (m)	Float position in steady state
*k* (-)	Number of points (vertices) used in the optimization procedure
*l_i_*	Lower bound for optimized parameter
*m* (kg)	Float mass
*n* (-)	Number of optimized parameters
*p*_1_ (Pa)	Pressure in the section where the float does not yet influence the flow
*p*_2_ (Pa)	Pressure in “vena contracta”
*Q* (m^3^/h)	Volumetric flow rate
*Q*′ (m^3^/h)	Additional volumetric flow rate due to movement of the float
*Q_max_* (m^3^/h)	Upper flow limit
*Q_min_* (m^3^/h)	Lower flow limit
*Q_ss_* (m^3^/h)	Volumetric flow rate in steady state
*r* (-)	Random number
*Re* (-)	Reynolds number
*t* (s)	Time
*t*_0.97_ (s)	Settling time
*u_i _*	Upper bound for optimized parameter
*u* (*x*)	Standard uncertainty
*u_c_* (*x*)	Combined uncertainty
*U*_95_ (*x*)	Expanded uncertainty at 95% confidence level
*U* (m/s)	Fluid velocity
*U*_1_ (m/s)	Fluid velocity in the section where the float does not yet influence the flow
*U*_2_ (m/s)	Fluid velocity in “vena contracta”
*U*′ (m/s)	Additional fluid velocity due to movement of the float
*V_f_* (m^3^)	Float volume
*x_i_*	Estimate of input quantity in a measurement process
*X_i_*	Input quantity in a measurement process
*y*	Estimate of output quantity in a measurement process
*Y*	Output quantity in a measurement process
*α* (-)	Reflection coefficient
*γ* (rad)	Tapering angle
Δ*h* (m)	Distance between *d_low_* and *d_high_*
Δ*x_i_*	Increment in the estimate of input quantity *x*_i_
Δ*y*	Increment in the estimate of output quantity *y* caused by Δ*x_i_*
*ν* (Pa·s)	Kinematic viscosity of the fluid
*ρ* (kg/m^3^)	Fluid density
*ρ_f_* (kg/m^3^)	Float density
*ω*	Optimized parameter

## Appendix A. Description of the Optimization Algorithm

The algorithm used in the article is a modified version of a simplex algorithm adapted for nonlinear constraint optimization problems. A set of parameters (*ω*_1_, *ω*_2_, …, *ω_n_*) that assures minimum of the objective function defined in Figure 4 is sought. Calculation of the objective function procedure is an external procedure from the point of view of the optimization algorithm.

At first five points (vertices) are randomly generated with the use of a formula:

(A1) $$\omega_{i}=l_{i}+r_{i}(u_{i}-l_{i})$$

where *r_i_* is a random number from the range 〈0,i) *l_i_*, *u_i_* are the lower and upper bounds for the optimization variable. If the generated set does not fulfill all of the constraints, a new set of points is generated.

The applied procedure operates on k≥n+5 points. Subsequent points are also generated with the use of a random numbers generator. If the generated point does not fulfill the functional constraints, it is shifted half a distance towards the center of gravity (CG) of the existing set of points, e.g., from point 9 to 9′. Such a procedure is repeated till the point is located in the acceptable area.

The objective function is calculated in each node of the polygon formed by all the randomly generated points. After generating *k* points the point with the maximum (the worst) value of the objective function (e.g., point 5) is replaced by an another one. This point is placed on a straight line crossing this worst point and the CG, on the opposite side of CG. The distance between the new point (e.g., 10 in the Figure A1) and CG is equal to the distance between the worst point and CG multiplied by a reflection coefficient *α* > 1.

If this point does not fulfill the hard constraints, it is shifted towards the acceptable area, at some distance *ε* from the border. If the point does not fulfill the functional constraints, it is shifted towards the CG by half the distance between this point and the CG. This procedure is repeated till the point reaches an acceptable area.

Introduction of the reflection coefficient *α* > 1 causes continuous growth of the area covered by simplex. It compensates the reduction of this area caused by the procedure of halving the distance between point lying outside the acceptable area and the GG. It makes it possible to find the minimum of the objective function even if the start points lie at a great distance from the optimal point. This way simplex does not collapse into a subspace.

The optimization procedure stops when five objective function values remain within a specified error band. After the tests of the algorithm it has turned out that *k* should lie in the range of (15–25). For *k* less than 10 it has been difficult to find the optimum and for *k* > 25 the calculation time has been unnecessarily prolonged.

## Appendix B. Uncertainty Evaluation

### Appendix B.1. Uncertainty of Calculations

An assessment of uncertainty of dynamic phenomena would be very difficult, if not impossible. Therefore, the analysis of uncertainty for steady state has been developed under assumption that the results will be comparable to transient.

According to Ref. [20,21], if the functional relationship between the input quantities *X*_1_, *X*_2_, …, *X_N_* and output quantity *Y* in a measurement process is:

(A2) $$Y=f\left(X_{1},X_{2},\ldots ,X_{N}\right)$$

then an estimate of *Y* denoted by *y* is obtained from Equation (A2) using input estimates *x*_1_, *x*_2_, …, *x_N_:*

(A3) $$y=f\left(x_{1},x_{2},\ldots ,x_{N}\right)$$

For uncorrelated input quantities *X_i_* the combined uncertainty may be found by combining the uncertainty of each of the contributing factors:

(A4) $$u_{c}(y)=\sqrt{\sum_{i=1}^{N}{\left(c_{i}u\left(x_{i}\right)\right)}^{2}}$$

where *u*(*x_i_*) are individual components of uncertainty and *c_i_* are sensitivity coefficients.

In VA meter analysis the output quantity is float elevation *h*. This applies both to the analytical and the experimental case. The basic functional relationship is a slightly transformed Equation (7):

(A5) $$Q=C\left(Re\right)A\left(h\right)\sqrt{\frac{2gm}{\rho A_{f}}}$$

where:

(A6) $$A\left(h\right)=\pi \left(hd_{f}\tan\gamma +h^{2}\tan^{2}\gamma \right)$$

Sensitivity coefficients *c_i_* are defined as the ratio of change of the output quantity *y* with respect to the input quantity *x_i_*. If an explicit formula for the output quantity is given, values of sensitivity coefficients are obtained by partial differentiation. In the analyzed case an another method proposed in Ref. [21] is applied. Sensitivity coefficients are obtained numerically, by calculating the effect of a small change in the input variable on the output. Output value *y* is first calculated using *x_i_*, and then recalculated using (*x_i_* + Δ*x_i_*), where Δ*x_i_* is a small increment in *x_i_*. The result can be written as (*y* + Δ*y*), where Δ*y* is the increment in *y* caused by Δ*x_i_*. Sensitivity coefficients are then calculated as:

(A7) $$c_{i}\approx \frac{\Delta y}{\Delta x_{i}}$$

A functional relationship between the input quantities and the output quantity *h* can be written, taking into account Equations (A5) and (A6), as:

(A8) $$f\left(h\right)=C\left(Re\right)\pi \left(hd_{f}\tan\gamma +h^{2}\tan^{2}\gamma \right)\sqrt{\frac{2gm}{\rho A_{f}}}-Q$$

Then the values of *h* can be calculated as the roots of the Equation (A8) with the use of standard numerical methods. Exemplary calculations of sensitivity coefficients for VA meter (3–30) dm^3^/h and flow rate step 10 dm^3^/h are presented in the Table A1.

**Table A1:** Calculation of the sensitivity coefficients *c_i_* for VA meter (3–30) dm^3^/h. The values in bold denote parameters that are incremented in a given row.

*x_i_*	Δ*x_i_*	*d_f_* in mm	*d_high_* in mm	*d_low_* in mm	Δ*h* in mm	*m* in g∙10^−2^	*ρ* in kg/m^3^	*υ* in m^2^/s·10^−5^	*Q* in dm^3^/h	*C*	*h* increment in m	*c_i_*
*d_f_*	0.01	**4.00**	4.31	4.08	236	5.38	1.293	1.45	0.01	0.2905	0.00014	0.014
*d_h_*	0.01	3.99	**4.32**	4.08	236	5.38	1.293	1.45	0.01	0.2905	−0.00791	−0.791
*d_l_*	0.01	3.99	4.31	**4.09**	236	5.38	1.293	1.45	0.01	0.2905	0.00876	0.876
Δ*h*	1	3.99	4.31	4.08	**237**	5.38	1.293	1.45	0.01	0.2905	0.0008	0.0008
*m*	0.01	3.99	4.31	4.08	236	**5.39**	1.293	1.45	0.01	0.2905	−0.00172	−0.172
*ρ*	0.01	3.99	4.31	4.08	236	5.38	**1.303**	1.45	0.01	0.2905	0.00072	0.072
*ν*	0.01	3.99	4.31	4.08	236	5.38	1.293	**1.46**	0.01	0.2905	0.00075	0.075
*Q*	0.1	3.99	4.31	4.08	236	5.38	1.293	1.45	**10.1**	0.2905	0.0079	7.9
*C*	0.01	3.99	4.31	4.08	236	5.38	1.293	1.45	0.01	**0.3005**	−0.00629	−0.629

After the assessment of all the uncertainty components according to guidelines from Ref. [20], the uncertainty budget was established (Table A2). During the uncertainty assessment the following factors have been taken into account. Type B uncertainty evaluation was used based on manufacturers’ declarations for *d_f_* (micrometer), *d_high_* and *d_low_* (stick micrometer), Δ*h* (caliper) and *m* (scales). For *ρ*, *υ* uncertainty of tabular data was used. For *Q* uncertainty of bell prover was used and for experimentally obtained *C* uncertainty was assessed using the uncertainty of measurement instruments and the goodness-of-fit of the approximating function (23).

**Table A2:** Uncertainty budget for VA meter (3–30) dm^3^/h.

Input Quantity x*_i_*	Nominal Value	Standard Uncertainty *u*(*x_i_*)	Unit	Sensitivity Coefficient *c_i_*	Unit	(*c_i_·u*(*x_i_*))^2^ in m^2^
*d_f_*	3.99 × 10^−3^	0.0075	mm	0.014	m/mm	1.1 × 10^−8^
*d_high_*	4.31 × 10^−3^	0.0075	m	−0.791	m/mm	3.52 × 10^−5^
*d_low_*	4.08 × 10^−3^	0.0075	m	0.876	m/mm	4.32 × 10^−5^
Δ*h*	236 × 10^−3^	0.5	m	0.0008	m/mm	1.6 × 10^−7^
*m*	5.38 × 10^−3^	0.001	kg	−0.172	m/kg	2.96 × 10^−8^
*ρ*	1.293	0.0025	kg/m^3^	0.072	m/(kg/m^3^)	3.24 × 10^−8^
*ν*	1.25	0.02	m^2^/s × 10^−5^	0.075	m/(m^2^/s × 10^−5)^	2.25 × 10^−12^
*Q*	0.01	0.0000125	m^3^/h	7.9	m/(m^3^/h)	9.75 × 10^−9^
*C*	0.2905	0.0025	-	−0.629	m	2.47 × 10^−6^
				*Σ(c_i_u(x_i_))^2^*	m^2^	8.11 × 10^−5^

Finally, the combined uncertainty equals:

(A9) $$u_{c}(h)=\sqrt{\sum_{i=1}^{N}{\left(c_{i}u\left(x_{i}\right)\right)}^{2}}=9.01\;\mathrm{mm}$$

and the expanded uncertainty is equal to:

(A10) $$U_{95}(h)=2u_{c}\left(h\right)=18.022\;\mathrm{mm}\;$$

Similar results were obtained for other flowmeters. The uncertainty was smaller for larger VA meters, e.g., for VA meter no. 5 for *Q* = 975 dm^3^/h *U_95_*(*h*) = 1.98 mm, for *Q* = 1500 dm^3^/h *U_95_*(*h*) = 2.77 mm and for VA meter no. 7 *Q* = 1381 dm^3^/h *U_95_*(*h*) = 4.46 mm.

### Appendix B.2. Uncertainty of Experiments

To assess the uncertainty of experiments, eight persons were asked to measure the float position in selected frames of a digitized film. The standard uncertainties lie in the range of (0.22–1.3) mm, so the expanded uncertainty is in the range (0.44–2.6) mm.

In the worst case the uncertainty of float position (including uncertainty of experiments and calculations) was equal to $\sqrt{18^{2}+2.6^{2}}=\;18.2\;\mathrm{mm}$.

## References

[1] Martin J.J. (1949). Calibration of rotameters. Chem. Eng. Prog..

[2] Lutz K. (1959). Die Berechnung des Schwebekörper-Durchflußmessers. Regelungstechn.

[3] Shafer M.R., Van Lone B. (1951). Correcting for Density and Viscosity of Incompressible Fluids in Float-Type Flowmeters. Res. NBS.

[4] Jiang W., Zhang T., Xu Y., Wang H., Guo X., Lei J., Sang P. (2016). The effects of fluid viscosity on the orifice rotameter. Meas. Sci. Rev..

[5] Singh S.N., Gandhi B.K., Seshadri V., Chauhan V.S. (2004). Design of a bluff body for development of variable area orifice-meter. Flow Meas. Instrum..

[6] Mandal N., Kumar B., Sarkar R., Bera S.C. (2014). Design of a flow transmitter using an improved inductance bridge network and rotameter as sensor. IEEE Trans. Instrum. Meas..

[7] Ahn H.J., Kim K.R. (2014). 2D Hall sensor array for measuring the position of a magnet matrix. Int. J. Precis. Eng. Manuf. Technol..

[8] Garcia-Diego F.-J., Sánchez-Quinche A., Merello P., Beltrán P., Peris C. (2013). Array of hall effect sensors for linear positioning of a magnet independently of its strength variation. A case study: Monitoring milk yield during milking in goats. Sensors.

[9] Mo B., Jiang F.Y., Zhang H.X. (2013). The Electronic Part of the Metal Tube Rotameter. Appl. Mechan. Mater..

[10] Liu C.Y., Lua A.C., Chan W.K., Wong Y.W. (1995). Theoretical and experimental investigations of a capacitance variable area flowmeter. Trans. Inst. Meas. Control.

[11] Baker R.C., Sorbie I. (2001). A review of the impact of component variation in the manufacturing process on variable area (VA) flowmeter performance. Flow Meas. Instrum..

[12] Baker R.C. (2004). The impact of component variation in the manufacturing process on variable area (VA) flowmeter performance. Flow Meas. Instrum..

[13] Ligeza P. (2018). Model and Simulation Studies of the Method for Optimization of Dynamic Properties of Tachometric Anemometers. Sensors.

[14] Harrison G.S., Armstrong W.D. (1960). The frequency response of rotameters. Chem. Eng. Sci..

[15] Dijstelbergen H.H. (1964). Rotameter dynamics. Chem. Eng. Sci..

[16] Ury J.F. (1963). The Pulsation Error of Rotameter. Isr. J. Teh..

[17] International Organization for Standardization (2018). Measurement of Fluid Flow in Closed Conduits. Guidelines on the Effects of Flow Pulsations on Flow-Measurement Instruments.

[18] Turkowski M., Szufleński P. (2013). New criteria for the experimental validation of CFD simulations. Flow Meas. Instrum..

[19] Korobiichuk I., Bezvesilna O., Ilchenko A., Shadura V., Michał N., Szewczyk R. (2015). A mathematical model of the thermo-anemometric flowmeter. Sensors.

[20] JCfGi M. Evaluation of Measurement Data—Guide to the Expression of Uncertainty in Measurement. https://www.bipm.org/utils/common/documents/jcgm/JCGM_100_2008_E.pdf.

[21] International Organization for Standardization (2005). Measurement of Fluid Flow—Procedures for the Evaluation of Uncertainties.

